# Feasibility of HIV self-test implementation among Mizo youths: a field investigation from Northeast India bordering Myanmar

**DOI:** 10.3389/fpubh.2025.1408990

**Published:** 2025-02-05

**Authors:** Amrita Rao, Henry Zodinliana Pachuau, Samiran Panda, Richard L. Chawngthu, Rita Zomuanpuii, Pranoti Hemade, Amit Nirmalkar

**Affiliations:** ^1^Indian Council of Medical Research - National Institute of Translational Virology and AIDS Research (NITVAR), Pune, India; ^2^Department of Social Work, Mizoram University, Aizawl, India; ^3^Indian Council of Medical Research Headquarters, New Delhi, India; ^4^Mizoram State AIDS Control Society, Aizawl, India

**Keywords:** HIV self-test (HIVST), Mizoram, youths, implementation-research, feasibility

## Abstract

**Introduction:**

This study investigated the potential of HIV self-test (HIVST) to reach individuals who otherwise might not access testing or antiretroviral therapy (ART). The study had two main objectives: (a) to develop an HIV self-test implementation plan based on the findings from qualitative inquiries with local stakeholders and (b) to examine HIVST uptake among youths in the urban setting of Aizawl district in Mizoram.

**Methodology:**

In the first phase, qualitative in-depth interviews (IDI) were conducted with HIV program officials, religious leaders, community influencers, youths, and key population groups. These inquiries guided the planning of strategic communication, community engagement, HIVST delivery, and linkages with HIV confirmatory testing services in phase two. Factors associated with the non-uptake of HIV confirmatory tests by youths following HIVST were analyzed quantitatively. Additionally, secondary data collected from attendees of the “Integrated Counselling and Testing Centre” (ICTC) were also analyzed.

**Results:**

The in-depth interviews underscored the need to introduce HIVST among Mizo youths. The respondents emphasized the importance of diverse outreach approaches and communication strategies, including the use of social media platforms, as critical components for successful HIVST implementation. They also provided valuable insights on the optimal locations and methods for making HIVST kits accessible. Among the youths who used HIVST, the majority were first-time testers (1,772/2,101; 84.3%). Those diagnosed with an undiagnosed HIV infection were started on ART. The preference for the blood-based HIVST format (1,162/2101; 55%) was noted to be slightly higher than the saliva-based format. Confirmatory test uptake was significantly higher among those with sero-reactive HIVST results (*χ*^2^ 23.89; *p* < 0.001). Factors independently associated with (adjusted odds ratio; AOR with 95% CI) “no-show for HIV confirmatory tests,” which hold significant programmatic implications, included “age > 20 years (1.47; 1.18–1.82),” “gender (men)” (1.25; 1.01–1.55), “education below 10th standard” (5.16; 2.66–10.01), “no prior HIV testing experience” (2.12; 1.61–2.81), and “unwillingness to undergo HIV confirmatory testing” (2.85; 2.05–3.96). Individuals who opted for the blood-based HIVST were 23% less likely (AOR 0.77; 95% CI; 0.62–0.96) to drop out of the HIV confirmatory testing process. Additionally, only 1% of respondents perceived HIVST as having self-harm potential.

**Conclusion:**

Sustained community engagement, effective networking with HIV program officials, and strategic communication were three critical pillars supporting the successful implementation of HIVST. There was a significant increase in HIVST uptake among young first-time testers.

## Introduction

Early diagnosis of HIV can trigger a cascade of events, starting with prompt treatment and ultimately leading to a reduction in HIV transmission (1). Globally, all countries are striving to achieve the 95:95:95 target (2) by 2025. The goals are that at least 95% of people living with HIV (PLHIV) would know their HIV status, 95% of those diagnosed would be on anti-retroviral treatment (ART), and 95% of those on ART would attain adequate viral load suppression in their blood and other body fluids. Achieving these targets contributes to the interruption of HIV transmission. Different strategies have been implemented in combination by various countries, such as provider-initiated testing, community-based test initiatives, partner notification, and mobile outreach, to increase HIV test uptake (3). Despite these initiatives, there continues to be a considerable gap in HIV test uptake. Globally, 86% of PLHIV are aware of their HIV status (4), with marked differences at the country level.

Similar to other countries, India has also implemented strategies over the past two decades to ramp up HIV test uptake. Presently, 77% of the PLHIV in India know their HIV status, of whom 84% are on ART, and 85% of those who are on ART have achieved adequate viral suppression. There is a gap of nearly 18% in terms of attaining the “first 95” (5). It is also important to note that variations exist in attaining these achievements across different states and districts within India, as the national average masks state-level heterogeneity. For instance, Mizoram, one of the northeastern states bordering Myanmar, is currently witnessing a generalized HIV epidemic with a very high prevalence among female sex workers (FSW) and people who inject drugs (PWID) at 24.7% and 19.8%, respectively (6). Only 64% of the estimated PLHIV are aware of their HIV status (5) in Mizoram. In this context, HIV self-testing stands out due to its attractive and pragmatic public health profile.

Based on systematic reviews, meta-analyses, and other primary research data, the World Health Organization (WHO) updated HIV self-test (HIVST) guidelines in 2019, which were first released in 2016 (7). HIV self-testing is a process in which a person undertakes the test with a blood or saliva specimen on their own and interprets the results either on their own in private settings or in the presence of someone they trust. Since then, various countries have adopted HIV self-testing and established their respective policies in place. In the meantime, studies across India have explored issues around the acceptability of HIV self-tests among different population groups. Research involving men having sex with men, pregnant women, truck drivers, and youths indicates that HIV self-tests, if available, would be acceptable and encourage people to know their own HIV status and seek further help (8–10).

Importantly, youths in India constitute nearly one-fourth of the entire population, and HIV prevalence in some of the states, including Mizoram, is of serious concern (5). While exploring such a situation in Mizoram, a rapid review emphasized the changing socio-economic conditions and geo-environmental factors. The existence of a porous international border that facilitates drug trafficking and unsafe sexual and injecting practices has increased the vulnerability of Mizo youths to HIV (11). The “*Technical Report 2021*,” published by the National AIDS Control Organization (NACO) in India, highlighted that the estimated HIV burden among young people (15–24 years) was highest in the states of Maharashtra, Andhra Pradesh, Karnataka, Uttar Pradesh, and Bihar, with figures ranging from 15,000 to 25,000 cases. In smaller states with much smaller population sizes, such as Manipur, Mizoram, Assam, Nagaland, Jharkhand, and Meghalaya, the estimated HIV burden varied between 1,500 and 3,000 cases (12). Moreover, the latest publication from NACO underlined the non-functional nature of the Adolescent Education Program in Mizoram (13), which includes topics related to HIV and sexual health.

Against this background, we conducted the present implementation research in Mizoram on HIV self-testing among the youths in the urban settings of Aizawl, the capital city and district of Mizoram. The overall purpose of this investigation was 2-fold: (a) to develop an HIV self-test implementation plan based on the findings of qualitative inquiry involving the local stakeholders (key informants) and (b) to examine HIV self-test uptake among youths in the urban setting of Aizawl following community-based execution of the intervention in collaboration with the State-AIDS Program. Qualitative and quantitative inquiry techniques were employed sequentially to fulfill these objectives.

## Methodology

This study was approved by the institutional ethics committee of the Indian Council of Medical Research (ICMR) - National Institute of Translational Virology and AIDS Research (NITVAR), formerly ICMR- National AIDS Research Institute (NARI) and Mizoram University. With the help of resource persons from the Mizoram State AIDS Control Society (MSACS) and Mizoram University, onsite field-level training and multiple online orientations were conducted with the project team members. A stepwise demonstration of “how to use an HIV self-test kit” was part of this training. The project operationalization plan was also finalized through participatory discussion during these training and orientation sessions. This process helped in deciding upon the access points for HIV self-test kits.

### Study design: two phases

This study was conducted in two sequential phases, namely qualitative inquiry, and quantitative investigation.

#### First phase: key informant interviews

In this phase, a qualitative inquiry was conducted with policy and program officials, religious and community leaders, HIV/addiction treatment service providers, youths, school dropouts, and participants from key population groups from the Aizawl district of Mizoram. The domains explored through in-depth interviews (IDIs) were “HIV situation in Mizoram,” “vulnerability to HIV,” “availability of HIV prevention and care services,” “HIV self-test,” and “implementation nuances around HIV self-test.” These IDIs, conducted from October through December 2022, helped attain saturation points (14) on explored domains. The findings were compiled based on the consolidated criteria for reporting qualitative studies (15) and informed the subsequent implementation plan in phase two.

#### Participants in the first phase

The study was located in the urban area of Aizawl, the most populous district of Mizoram, with an HIV prevalence among the general population being >1% (16). Participants for qualitative interviews in this phase were selected purposively to capture a broader understanding of the community. Interactions were held with the professors and principals in universities and colleges, church and community leaders, youth leaders from the Young Mizo Association (YMA), and superintendents of the local hospitals and rehabilitation homes. Such interactions were conducted by the principal investigators and project staff over 2 months. All of the participants were adults (above 18 years of age). Written informed consent was obtained from each IDI participant before the interviews were audio recorded. This formative work facilitated planning activities in different *vengs* (localities) of Aizawl, which will be undertaken in the next phase.

#### Second phase - implementation

During the second phase, the project staff developed “Information, Education, and Communication (IEC)” materials for the awareness campaign in the form of pamphlets, posters (examples in Annexure 1), and videos in consultation with the local stakeholders. Subsequently, community-based awareness activities were planned through meetings with state-level partners and influencers in the community, such as church leaders, leaders for the Young Mizo Association, and NGOs from key population groups. This paved the path for implementing HIV self-test activities in the district of Aizawl. A short semi-structured interview was conducted with the youths belonging to the age group 18–24 years who undertook HIV self-tests, which are presented below based on the guidelines for reporting observational studies (17).

#### Activities in the second phase

Based on the findings of phase one, outreach sites were set up in different community locations with the engagement of youths. Youths were encouraged through community-based strategic communication events to access HIV self-tests from designated outlets.

### Data analyses

Qualitative interviews in the first phase were conducted by a team comprising a research assistant (moderator) and a multitasking project staff member (note taker). They were from a social science background and had experience in applying qualitative research methods.

The guides and probes used in IDIs were pilot-tested and modified as appropriate for local socio-cultural relevance and ease of understanding. Interviews were conducted in the Mizo language, with which the participants were most comfortable. Interviewing sites were selected at the participants’ convenience to ensure privacy and confidentiality. The audio-recorded interviews were transcribed verbatim and later translated into English and were coded using N. Vivo version 11 (QSR International, Melbourne, Australia) for thematic content analysis.

In the second phase of the study (from December 2022 to June 2023), youths undertaking HIV self-tests (assisted or unassisted) and completing a short semi-structured interview were followed up telephonically thrice; after 1 week, 1 month, and 3 months (as needed) to help establish linkages with the “Integrated Counselling and Testing Centre” (ICTC) for the HIV confirmatory test and were compensated for the required travel. Descriptive statistics were used to present the profile of the youths. Distributions of the outcome and independent variables for normality were examined through the Shapiro–Wilk test. Upon fulfilling the normal distribution of the variables, univariate and multivariate analyses were conducted to identify factors associated with the dependent variable, “youths not undertaking confirmatory HIV tests after an HIV self-test.” This dependent variable was of interest because of its potential to identify areas where the State-AIDS program could have an intervention focus. The analysis was conducted using Microsoft Excel (Microsoft Corp., USA) and the Statistical Package for the Social Sciences (SPSS) Version 15.0 (SPSS for Windows, IBM®, USA).

Directly observed implementation processes were documented in detail to generate an in-depth understanding. Secondary data on ICTC attendees and their HIV seroreactivity were also analyzed.

## Results

### In-depth interviews during qualitative inquiry under the first phase

During the first phase, 41 IDIs were conducted with various stakeholders, including the State HIV policy and program officials (six), service providers at ICTC and Anti-Retroviral Treatment (ART) centers (seven), Opioid Substitution Treatment (OST) center staff (three) and Targeted Intervention (TI) site staff (Five). The other IDI participants were as follows: seven church leaders or community influencers, eight local youths or youth leaders, three members of a PWID group, and two people living with HIV (PLHIV). Each interview lasted 45–60 min, and the participants were aged 19–65 years. Of the 41 participants, 23 were men, and 18 were women. Four of them had studied till 12th standard, while the rest completed graduation or higher levels of education. Most participants were involved in income-generation activities except one who was a student and had no income. The following themes emerged through content analysis:

#### Need for HIVST

The necessity for increasing HIV test uptake was underlined as some pockets in the community were perceived as “yet unreached” by the State-AIDS program. In this context, HIV self-test (HIVST) was considered beneficial to those who were still hesitant to access HIV test centers. Early detection was viewed as an important first step toward early treatment.

*“It is not difficult to take an HIV test, but some people are afraid of getting tested…there are many who do not want to have the test because they fear stigma.”*

- 24 years, male, PWID.

“*Self-testing is a good thing. Currently, it is difficult to reach the 95 target. If self-test [becomes] available, I think we will reach our first 95 goals.”*

- 44 years, female, Program Official.

Most of the participants felt that the HIVST should be introduced between the age group of 18 to 24 years and a few thought that it could even be introduced at an age as young as 12 years.

“*Age group 18–24 is very good…most people finish their high school education and become college students at the age of 18…[however] students of these days become sexually active very early.”*

- 33 years, female, Program Officer.

#### Suggested means of reaching out

IDI participants emphasized the need for awareness around HIVST, and newspaper, television, and social media platforms were suggested means of communication. It was perceived that the engagement of celebrities or influencers would play a key role in creating awareness.

*“Most effective way would be letting the popular celebrities and influencers raise and promote awareness…since it could be difficult to gather youths separately, it would be great to give the awareness in the Church or maybe even organize events that include people they admire.*”

- 30 years, male, Medical Officer – ART center.

“*An advertisement on TV might be good…radio can reach the countryside…it can be placed at the bottom of the front page of the newspaper regularly…so, in addition to giving awareness in the community, especially in the church, I think it can be done in this way.”*

- 65 years, Women, Member of Women’s Group.

In addition to urban settings, schools and colleges in rural areas were considered sites where awareness campaigns on HIVST were perceived as needed. Participants felt that most youths would prefer saliva-based kits over blood due to fear of blood draw. Non-governmental organizations (NGOs) were considered important partners for distributing HIV self-test kits; clear instructions on the kits on “how to do” it in the local language were flagged as important. Most participants said they would prefer someone to assist them during the test.

### Community outreach in the second phase before the quantitative investigation

Based on the inputs received during IDIs, IEC materials such as pamphlets, posters, and video clippings were initially developed by local boys and girls, resource persons, and healthcare workers in English and later translated into the Mizo language (both used in the community). These were pilot-tested among the youths and public health experts before finalization. Some IEC materials were disseminated through social media platforms such as WhatsApp groups and Instagram. Awareness campaign activities were conducted in different *vengs* (localities) of the Aizawl district with the help of youth leaders from the Young Mizo Association (YMA), Church-leaders and influencers associated with community-based organizations, rehabilitation homes, universities, and hospitals. During the Winter Fest activities held at Mizoram University and other colleges, Red Ribbon Clubs (RRC) facilitated the event. Local resource persons were involved, and space was provided for the engagement of the youths in discussions around HIV and HIVST.

Pamphlets developed as part of this study were distributed to the attendees of the awareness campaigns held at different street corners. Furthermore, posters were displayed at strategic locations where youths assembled, and the project staff set up HIVST stalls at some of these sites to encourage youths to drop by. Table 1 shows that the uptake of HIVST varied between different delivery points, the highest uptake being observed at the stalls organized by key population groups, YMA, and healthcare facilities. Notably, a lower proportion of youths at college, YMA, church, and *veng-based* stalls reported previous HIV testing experience, ranging from 6 to 20%.

**Table 1:** Outreach sites and HIV test uptake.

Sites	Number of events	Total number of attendees at the events	Uptake of HIVself-test at the campaign sites by youths (%) and socio-demographic profile of youth attendees
Colleges/University	7	6,590	n1 = 869 (13.2%) Men: 362 (41.7%) Women: 507 (58.3%) Mean age: 20.79 years (SD 1.62 years) Median age: 21 years (20–22) *Participants’ residential district* Aizawl: 457 (52.7%) Non Aizawl: 410 (47.3%)
Stalls with Young Mizo Association (YMA)	6	600	n2 = 596 (99.3%) Men 330 (55.4%) Women 266 (44.6%) Mean age: 20.51 years (SD 1.77) Median age: 20 years (19–22) *Participants’ residential district* Aizawl: 450 (75.6%) Non Aizawl 145 (24.4%)
Churches	3	175	n3 = 101(57.7%) Men: 38 (37.6%) Women: 63 (62.4%) Mean age: 20.79 years (SD 2.05) Median age: 20 years (19–23) *Participants’ residential district* Aizawl: 100 (99%) Non Aizawl: 1 (1%)
*Veng-based* test site (facilitated by key population group)	12	269	n4 = 269 (100%) Men 190 (70.6%), women 79 (29.4%) Mean age: 21.71 years (SD2.07) Median age: 22 years (20–24) *Participants’ residential district* Aizawl: 103 (38.3%) Non Aizawl: 166 (61.7%)
Hospital-based stalls	3	198	n5 = 194 (97.8%) Men: 54 (27.8%) Women: 140 (72.2%) Mean age: 20.98 years (SD 1.73) Median age: years (20–22) *Participants’ residential district* Aizawl: 108 (55.7%) Non Aizawl: 86 (44.3%)
Rehabilitation centers	3	111	n6 = 72 (64.8%) Men: 60 (83.3%) Women 12 (16.7%) Mean age 21.86 years (SD 1.98) Median age: 22 years (20–23) *Participants’ residential district* Aizawl: 48 (66.7%) Non Aizawl: 24 (33.3%)

Additional media coverage through local newspapers as part of the World AIDS Day activity, discussion on local television channels, and dissemination of video clippings through the Mizoram University website and friend-to-friend forwards through WhatsApp helped create awareness.

### Participants’ profile in the second phase

During the second phase of this study (December 2022 to June 2023), HIVST was taken by 2,101 youths. Their mean age was 20.9 years (median 21, minimum 18, maximum 24, SD ±1.8 years); the proportion of men (1034/2101, 49.2%) and women (1,067/2101, 50.8%) were almost equal, and the majority of them were not married (1947/2101; 92.6%). While <1 % (0.7%, 15/2101) of the youths had no formal education, 37.9% (798/2101) had studied up to 12th standard, and 60% (1263/2101) had completed graduation or a higher level of education. Most of these youths (1266/2101; 60%) were from the district of Aizawl; the mean number of their family members was 5 (minimum 1, maximum 15, SD ± 1.88).

### Awareness around HIV and HIVST

Approximately 85% of the participants (1783/2101) were exposed to HIV awareness activities, and most believed these campaigns encouraged testing. Notwithstanding, a very high proportion (1772/2101; 84.3%) of the youths taking HIV self-tests under this project had never sought an HIV test earlier. Among those (318/2101; 15%) with previous HIV test experience, 37.1% (144/318) had taken it from the ICTC, and 11.3% each from targeted intervention (TI) sites (36/318) and private laboratories (36/318). Notably, more than half of the participating youths (1131/2101, 53.8%) were unaware of HIVST before the current project, which pointed toward a gap in the existing awareness program.

### Preferences and how-to-do

Preference for blood-based HIV self-tests (1162/2101; 55.3%) was higher among the youths than for saliva-based test kits. One-fourth of the youths undertaking HIV self-test preferred someone explaining the test and then seeking help if needed, while 73% of participants (1538/2101) wished someone to explain every step while taking a test. Social media was perceived as the suitable platform for creating awareness around HIVST by the majority (1546/2101; 73.6%), and the next preferred modality was the distribution of pamphlets and posters. While 97% of those taking HIVST (2034/2101) under the current project found it as an easy-to-use kit, only 3% (65/2101; 3%) had a contrary view.

### Perceptions around HIVST

When asked about the potential harm an HIV self-tests could cause to society, 27 out of 2101 youths (1.3%) considered this as a possibility, and 3.5% (74/2101) were unsure of such consequences – the rest did not perceive any such threat. Most of the youths (73.5%, 1525/2074) felt that each HIVST kit should be priced below INR 100 (approximately 1.2 US$), and 20% (418/2074) opined the price per kit could be more (INR 200 or more). Only 10 participants favored making these kits available free of cost.

### Linking participants to ICTC and ART centers

**Figure 1:**
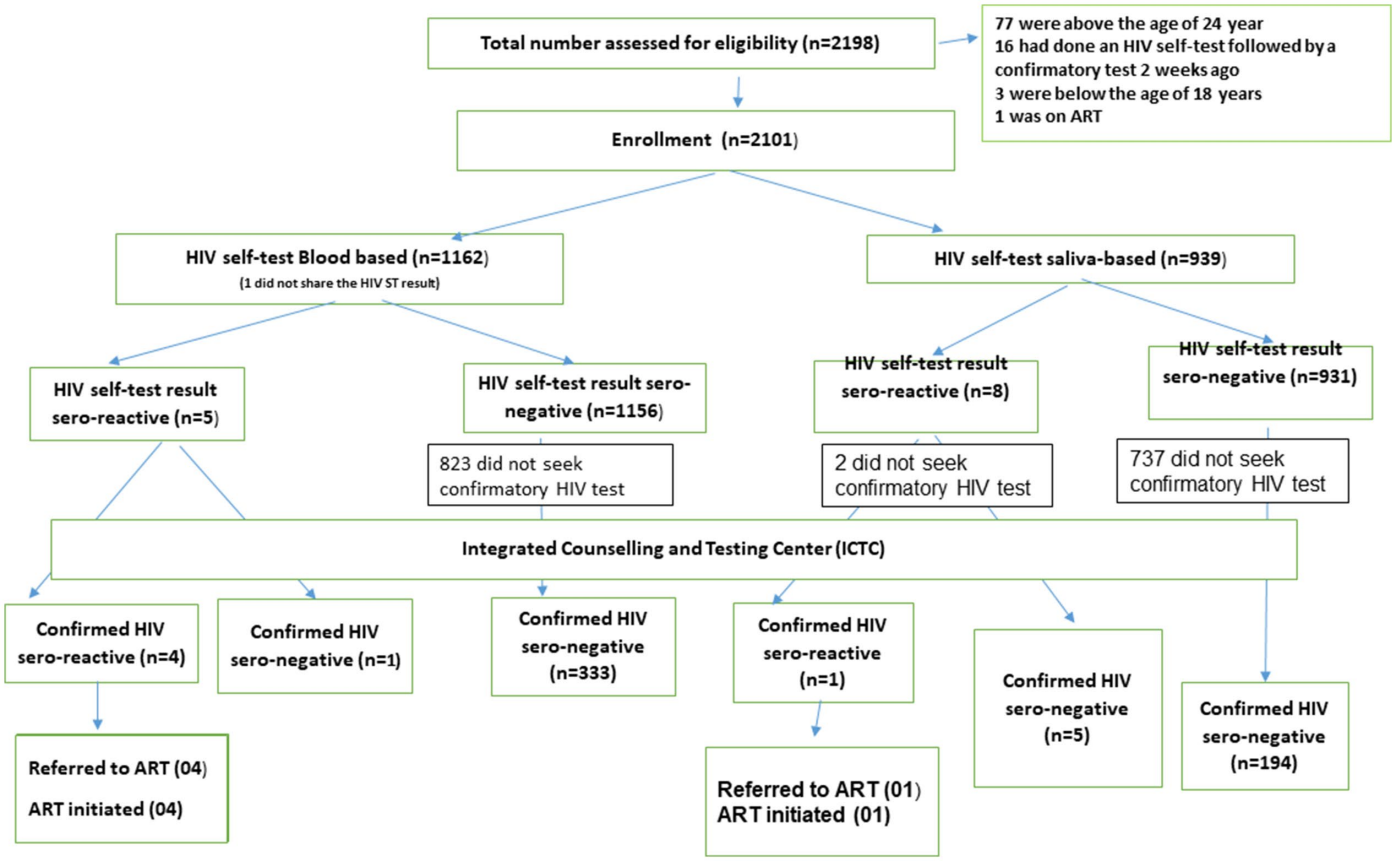
Flow diagram.

Of the 2,101 takers of the HIV self-test, 538 (25.6%) sought confirmatory tests. The proportion showing up for the HIV confirmatory test was significantly higher among those identified as HIV sero-reactive (11/13; 85%) compared to those who had a non-reactive self-test result (527/2087, 25%; *χ*^2^ 23.89; *p* < 0.001). Five participants were identified as HIV sero-reactive at the ICTC (Figure 1), and they were linked with ART centers and initiated on ART medication.

### Univariate analysis

As factors associated with “youths not undertaking an HIV confirmatory test (no-show) following HIVST” (dependent variable) have programmatic implications, univariate analysis was conducted to identify them. While “youths not taking an HIV confirmatory test following HIVST” was considered as the outcome (dependent variable), explanatory variables tested for their association were “age,” “gender,” “educational status,” “being in a relationship,” “preferred HIVST format (blood vs. saliva),” “prior HIV test experience,” “residential location,” “assisted vs. non-assisted HIVST,” “felt at ease with HIVST,” “expressed willingness to undergo an HIV confirmatory test,” and “perceived utility of HIVST as a component of HIV awareness campaign” (Table 2). Of the aforementioned explanatory variables, “residential location,” “assisted vs. non-assisted HIV ST,” and “felt at ease with HIVST” were not statistically significantly associated with the outcome variable. The rest of the variables had a statistically significant association with “non-uptake of HIV confirmatory test following HIVST” at a *p* < 0.05.

**Table 2:** Factors associated with no-show for HIV confirmatory test following HIVST in univariate analysis.

Participant’s characteristics	HIV confirmatory test undertaken		OR (95% CI)	*p*-value
	No 1563 (%)	Yes 538 (%)		
Age (in years)				
>20	897 (57.5)	250 (46.5)	1.56 (1.27–1.89)	.<0.01
<=20	664 (42.5)	288 (53.5)	Ref	
Gender				
Men	813 (52)	221 (41.1)	1.56 (1.28–1.89)	<0.01
Women	750 (48)	317 (58.9)	Ref	
Education				
Below 10th standard	220 (14.3)	12 (2.2)	7.23 (4.01–13.05)	<0.01
Above 10th standard	1,322 (85.7)	522 (97.8)	Ref	
Being in a relationship				
Ever been in a relationship	105 (6.9)	07 (1.4)	5.35 (2.47–11.58)	<0.01
Not been in a relationship	1,426 (93.1)	509 (98.6)	Ref	
Preferred HIVST format				
Blood-based	824 (52.7)	338 (62.8)	0.66 (0.54–0.81)	<0.01
Saliva-based	739 (47.3)	200 (37.2)	Ref	
Prior HIV test experience				
No	1,349 (86.9)	423 (78.8)	1.78 (1.38–2.29)	<0.01
Yes	204 (13.1)	114 (21.2)	Ref	
Residential location				
Aizawl	924 (59.2)	342 (63.6)	0.83 (0.68–1.02)	0.076
Non-Aizawl	636 (40.8)	196 (36.4)	Ref	
Assisted vs. non-assisted HIVST				
Assisted HIVST	1,429 (92.8)	489 (92.1)	1.10 (0.76–1.60)	0.594
Non-assisted HIVST	111 (7.2)	42 (7.9)	Ref	
Felt at ease with HIVST				
Yes	1,512 (96.8)	522 (97.2)	0.87 (0.48–1.56)	0.638
No	50 (3.2)	15 (2.8)	Ref	
Expressed willingness to undergo an HIV confirmatory test				
No	969 (62.8)	63 (11.9)	2.94 (2.20–3.92)	<0.01
Not sure	167 (10.8)	24 (4.5)	3.17 (2.04–4.94)	<0.01
Yes	406 (26.3)	442 (83.6)	Ref	
HIVST to be part of an awareness campaign				
No	131 (8.5)	28 (5.3)	1.67 (1.09–2.54)	0.05
Yes	1,409 (91.5)	504 (94.7)	Ref	

### Multivariate analysis

Age, gender, and residence location (due to their capability of adjusting for unrecorded confounders) and variables with programmatic relevance and having statistically significant association with the outcome variable in univariate analysis (at the level *p* < 0.1) were entered into a multivariate logistic regression model. Results of the multivariate model and computed adjusted odds ratios (AORs with their respective 95% confidence interval; CI) are presented in Table 3. Adjusted for eight explanatory variables (listed in Table 3), factors independently associated with “no-show for HIV confirmatory test” were as follows: “aged >20 years,” “gender (men),” “educational status (<10th standard),” “preferred HIVST format (blood/saliva-based kit),” “no prior HIV testing experience,” and “unwillingness to undergo HIV confirmatory testing.” Notably, individuals preferring blood-based HIVST were 23% less likely (adjusted OR 0.77; 95% CI; 0.62–0.96) to drop out (“no-show”) from HIV confirmatory tests.

**Table 3:** Factors independently associated with no-show for HIV confirmatory test following HIVST.

Variables	Adjusted odds ratio (AOR) (95% CI)	*p*-value
Age (in years)		
>20	1.47 (1.18–1.82)	<0.01
<=20	Ref	
Gender		
Men	1.25 (1.01–1.55)	<0.05
Women	Ref	
Education		
Below 10th standard	5.16 (2.66–10.01)	<0.01
Above 10th standard	Ref	
Being in a relationship		
Ever been in a relationship	1.93 (0.80–4.64)	0.14
Not been in a relationship	Ref	
Preferred HIVST format		
Blood-based	0.77 (0.62–0.96)	0.02
Saliva-based	Ref	
Prior HIV test experience		
No	2.12 (1.61–2.81)	<0.01
Yes	Ref	
Residential location		
Aizawl	0.92 (0.74–1.14)	0.44
Non-Aizawl	Ref	
Expressed willingness to undergo an HIV confirmatory test		
No	2.85 (2.05–3.96)	
Not sure	3.41 (2.14–4.44)	<0.01
Yes	Ref	<0.01

### Secondary data analysis

**Figure 2:**
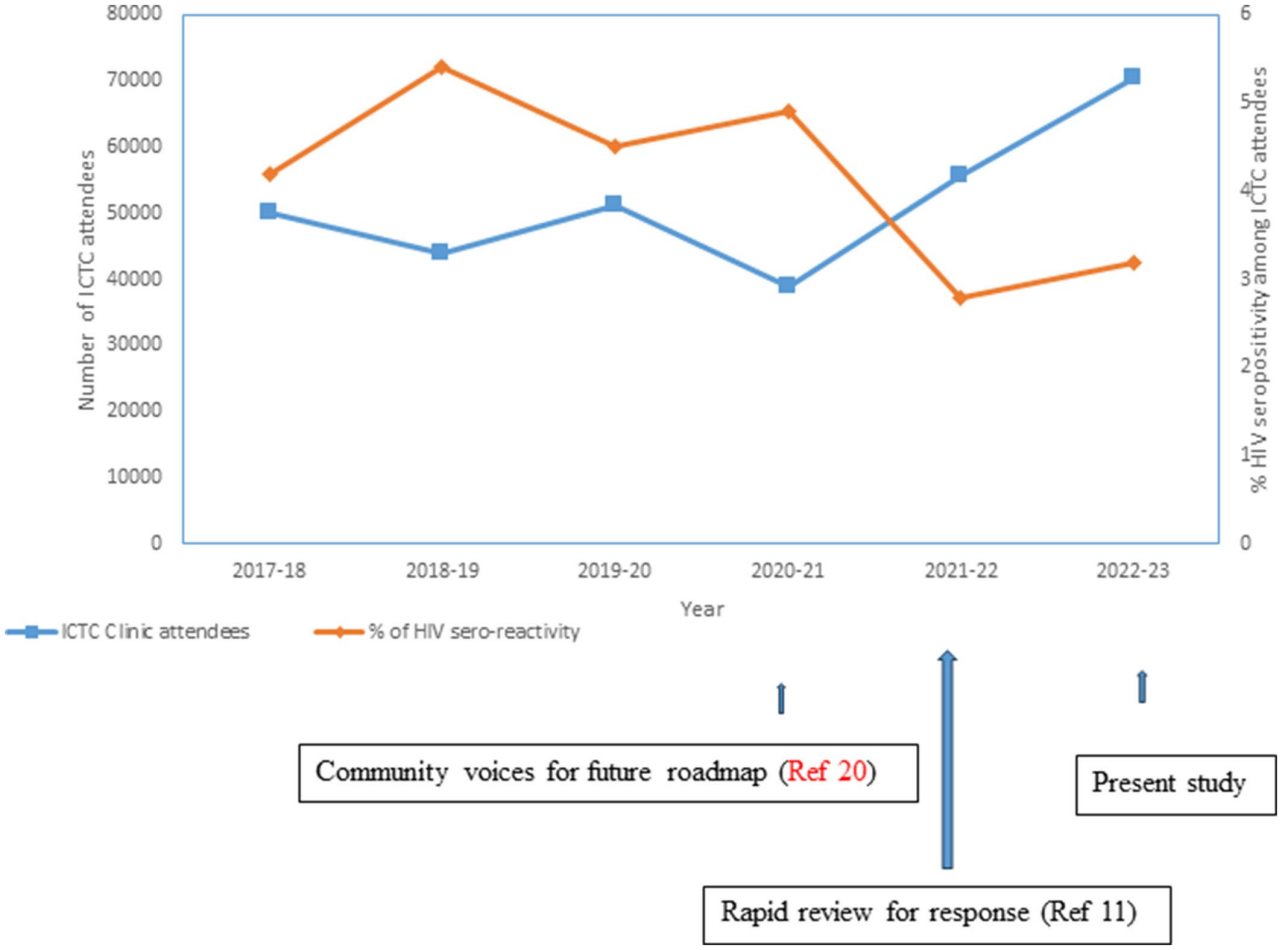
Change over time: integrated counselling & testing centre (ICTC).

While the immediate effect of the present implementation study was captured through the aforementioned primary data analysis, we analyzed aggregated secondary data made available in the public domain by NACO (5, 13, 18, 19). This helped in examining what happened outside the project setting (spin-off benefit), as Table 1 revealed that all who congregated at some of the event sites did not necessarily undergo HIVST, either due to unwillingness or not being in the “youth age category.” The attendance at ICTC centers in Mizoram was mapped with 2017–2018 as the base year. Figure 2 indicates a gradual dip in ICTC attendance along with concurrently low identification of people diagnosed with HIV, which was followed by an uptick in the number of people seeking HIV tests that crossed past the pre-2017 level. The upwardly looking arrows proximal to the X-axis in Figure 2 indicate relevant and sustained research initiatives focusing on community engagement (20), along with the review of the HIV epidemic situation and responses (11).

## Discussion

In many countries, HIVST is regarded as a useful public health tool in the bouquet of HIV prevention and treatment services. A recent systematic review and meta-analysis underlined HIVST as safe and effective among the general population in sub-Saharan Africa with a range of delivery options (21). The review recognized that HIVST linked additional people to HIV care. Such findings supported the wider availability of HIVST to reach those who might not otherwise access testing. Research on HIVST among different key population groups and youths across India (8–10) has correspondingly generated evidence of its acceptability. While the National and State AIDS Control Programs in the country are taking such global and in-country developments into cognizance, investigations on HIVST implementation in the real world that the program could draw upon have been sparse. Against this background, the present study generated a set of useful evidence by teasing out feasibility issues, providing details about HIVST implementation, and capturing measurable outputs achieved by establishing noteworthy and effective community linkages.

The focus of this inquiry was the feasibility of HIVST implementation among the youths of Aizawl, Mizoram. We drew upon deploying qualitative and quantitative techniques to achieve its objectives. The current burgeoning generalized HIV epidemic in Mizoram (11) had its beginning during the first detection of the virus in the neighboring northeastern state of Manipur in the early 1990s (22). This formed the epidemiological context of the present investigation. However, a rising footfall for HIV test uptake has been recorded at the ICTCs post-2021 in the State of Mizoram (5, 13, 18, 19) (Figure 2). The rapid expansion of HIV service is needed to achieve the 95–95–95 goal by 2025. The findings of this research provide crucial evidence to address programmatic gaps. Notably, qualitative responses presented above under the first phase of the current research resonated with this sense of urgency.

As highlighted by the process description and study outputs, a multi-prong outreach strategy guided by in-depth interview responses and stakeholder consultations made HIVST implementation feasible. This was in consonance with our earlier investigation in five high-HIV-burden districts of Mizoram, where HIV vulnerability, challenges of the existing prevention and care services, and required program elements were explored (20). We followed the leads emerging from this previous community-based investigation.

During the second phase of the present investigation, more than 2,000 youths undertook HIVST in about 6 months, highlighting the appropriateness of the adopted strategy. Notably, the uptake of HIVST was highest at the HIVST stalls organized by key population groups, YMA, and healthcare facilities. The community’s peer leaders and outreach workers played important roles in engaging the youths from the high-risk groups, youths from the YMA community, and those working at the healthcare facilities. The strategic communication around HIVST was youth-centric at all sites. However, the community leaders played a crucial role in the uptake of HIV self-tests. Similar approaches also facilitated the HIVST uptake in Nigeria (23). Although 85% of those undertaking HIVST reportedly were exposed to state-run HIV awareness campaigns, prior HIV test experience was present only in a few of them. This underlines the effectiveness of our strategic behavior change communication rather than the traditional awareness campaign. In alignment with the other researchers urging upon the necessity of bridging translational gaps and identifying priority areas for capacity building to strengthen community engagement (24), we ensured active community involvement as well as the participation of the State-AIDS program personnel at all stages of this implementation research. This approach helped ensure the development and delivery of culturally sensitive and acceptable interventions.

Studies have shown that preference for types of HIVST differs in different countries and population groups (25, 26); the way one wishes to go through a self-test (assisted/supervised, semi-assisted, or unassisted) also differs. For example, in one of the studies from the Democratic Republic of Congo, youths tended to lean toward unsupervised HIVST, particularly when they had prior knowledge about it (27). Contrastingly, more than half of the youths in Mizoram were not aware of HIVST before taking part in the present investigation, and approximately 90% of them opted for an assisted HIVST. Regarding such assistance, our findings were similar to those from Zimbabwe, where the youths articulated, “When healthcare workers are supportive, I would rather not test alone” (28).

Although a commonly held view during the qualitative inquiry in our setting indicated that individuals might prefer saliva-based HIVST over blood-based formats due to fear associated with blood drawing, during the quantitative investigation phase, a greater proportion of youths opted for blood-based HIVST (55 vs. 45%; Figure 1). Similar findings were revealed from a study conducted in Nigeria among young people aged 14–24 years (29). The programmatic inclusion of HIVST, however, needs to emphasize the role of self-test as a screening tool (irrespective of blood- or saliva-based test format) and the necessity of following it up with HIV confirmatory tests. Multivariate analysis revealed that the Mizo youths undertaking blood-based HIVST had a lesser likelihood of “no-show” for HIV confirmatory tests compared to those who preferred saliva-based tests.

A significant outcome of this research was the voluntary uptake of HIVST by a large number of youths in Aizawl in 6 months, along with the establishment of quick linkages (within 2 months) to HIV confirmatory test facilities and ART centers, as needed. The majority of the participants from the community with a sero-reactive HIVST result could be linked to ICTC and HIV care services (Figure 1). However, HIV-seronegative individuals were more reluctant to pursue confirmatory tests.

Multivariate analysis suggested that youths above 20 years of age, men, those with lower educational backgrounds, and individuals without prior HIV testing experience were less likely to seek HIV confirmatory tests. We, therefore, recommend prioritizing these factors when implementing HIVST under the State-AIDS program. A systematic review from six sub-Saharan African countries reported that 65–70% of men took 7–15 months to undergo a confirmatory test after a reactive HIV self-test (30). Similarly, in Zambia, those with no prior experience with HIV testing had lower chances of linking to a confirmatory test (31).

However, this study had some limitations. The approach used in this investigation may not be suitable for settings with limited availability of HIV prevention and care services. Moreover, the success of this intervention relied on supplementary human resources, albeit minimal, including investigators and field-level social workers skilled in qualitative data collection and analysis, community mobilization, and networking. Finally, the findings from this HIVST implementation are limited in their transferability. The study was conducted in a specific context, guided by a thorough analysis of state-specific issues related to the HIV epidemic and responses. Nonetheless, the process description presented here provides a valuable framework for frontline community groups, program managers, and researchers from other settings to collaborate and implement HIVST initiatives.

## Conclusion

In conclusion, this study provides valuable insights into the implementation of HIVST-based interventions in Mizoram. The success of this initiative highlights the importance of community engagement, strategic communication with youth, and active partnerships with HIV program officials and service providers. Adapting HIV self-testing for other settings and socio-cultural contexts will require tailored planning. This study was limited by its short follow-up period and its focus on the urban area of the Aizawl district in Mizoram, making it challenging to generalize the results across the country. However, the well-documented intervention provides important lessons that can inform similar efforts elsewhere. Additionally, the cost estimates generated from this research could support the replication of such initiatives in other settings. Finally, addressing barriers to confirmatory testing through socio-culturally appropriate interventions will be critical for sustaining progress in HIV containment in the long run.

## Data Availability

The raw data supporting the conclusions of this article will be made available by the authors without undue reservation.
