# Supplementary material for: Feasibility of HIV self-test implementation among Mizo youths: a field investigation from Northeast India bordering Myanmar
Source: Front Public Health. 2025 Feb 5;13:1408990. doi: 10.3389/fpubh.2025.1408990 (PMC11835815; doi:10.3389/fpubh.2025.1408990)
Supplement: Supplementary file 1 [file Supplementary_file_1.docx]

ANNEXURE I : Information education & communication strategies for interventions

PAMPHLETS FOR CREATING AWARENESS

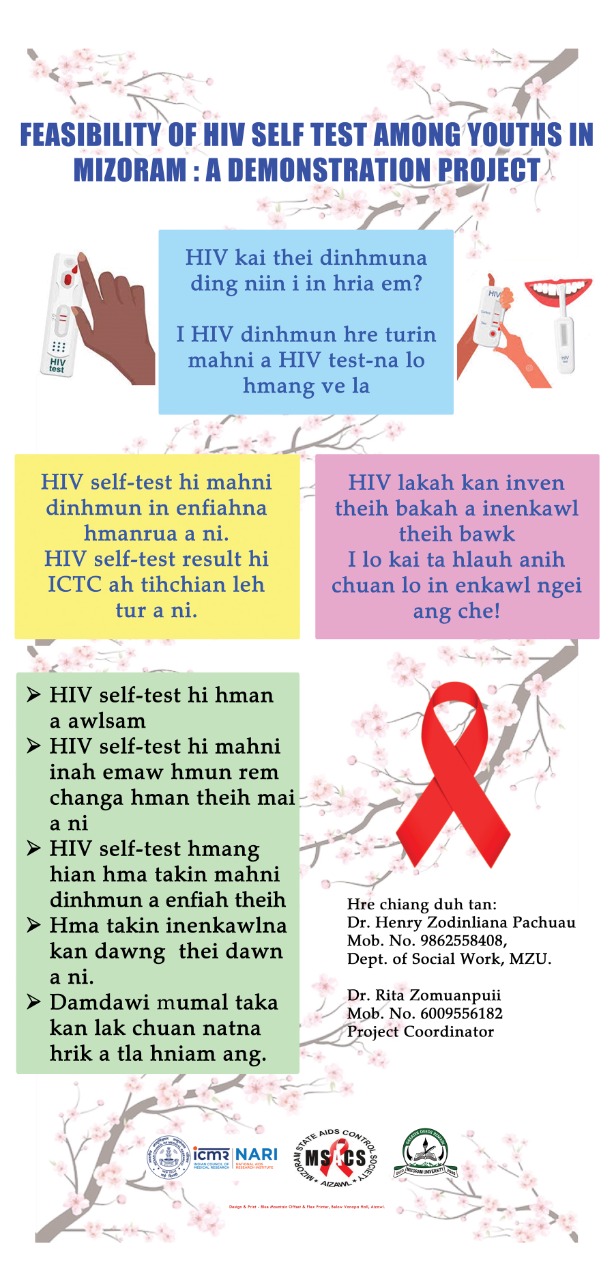


BANNER in Mizo language
